# A GIS-toolbox for a landscape structure based Wind Erosion Risk Assessment (WERA)^✰^

**DOI:** 10.1016/j.mex.2024.103006

**Published:** 2024-10-30

**Authors:** Roger Funk, Lidia Völker

**Affiliations:** Leibniz Centre for Agricultural Landscape Research (ZALF), WG Soil Erosion and Feedbacks, Eberswalder Str. 84, D-15374 Muencheberg, Germany

**Keywords:** Landscape elements, Element height, Wind shadow, Wind protection, Wind erosion, Wind Erosion Risk Assessment (WERA)

## Abstract

The landscape structure influences the local wind field by lowering the wind speed and thus reducing the wind erosion risk. An important parameter is the height of each landscape element, as this determines the length of wind protection behind it. Further determining parameters are the wind speeds above a threshold value for initiating wind erosion and the corresponding wind directions. The presented method combines heights of landscape elements and the directional transport capacities of erosive wind speeds to derive a map of the spatial wind speed reduction by landscape structures. This map can be combined with the soil-derived erodibility map to get finally a wind erosion risk map which includes landscape effects.

• The ArcGIS toolbox “WERA” allows a detailed analysis of the landscape structure on wind erosion processes,.
• The method allows both the identification of risk areas and needs for additional plantings of LE.
• The area-specific query determines the wind erosion risk for field blocks, districts or municipalities.

## Graphical abstract

**Figure:**
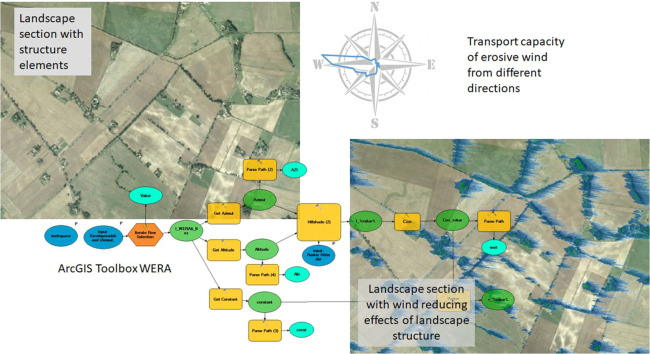

Specification table

**Table:** 

Subject area:	Environmental Science
More specific subject area:	Landscape structure
Name of your method:	Wind Erosion Risk Assessment (WERA)
Name and reference of original method:	Wind Erosion Prediction System (WEPS, USDA 2020); German Standard DIN 19706 [1]: Soil quality - Determination of the soil exposure risk from wind erosion
Resource availability:	Microsoft Excel 2013; ESRI ArcGIS 10.6.1, Spatial Analyst; ArcGISPro

## Background

Sandy soils and dry climatic conditions promote the processes of wind erosion particularly in Brandenburg. The problem has intensified considerably in the last 20 years, as climate change has become noticeable through increasingly prolonged dry periods and rising temperatures [2,3]. Since there is a high potential for wind erosion from the soils texture and the dry climatic conditions anyway, it are mainly the accompanying factors that ultimately decide whether wind erosion occurs on a field or not. One of these further more stable factors is the landscape structure, which includes all obstacles on a surface which can be assigned with a certain height, as forests, single trees and groups of them, hedges or buildings. The effect is the lowering of the surface wind speed at the place, but also far-reaching up to the 40-fold of their height in the leeward direction [4,5]. Landscape structure can thus contribute to a considerable reduction in the area at risk from wind erosion. In Brandenburg, this led to an approx. 60 per cent reduction of the area at very high wind erosion risk [6].

The German standard DIN 19706 is the obligatory basis for assessing wind erosion for administrative purposes in Germany. However, it does not contain any methodology for taking the direction-dependent wind effect into account. The aim of the presented method is to remedy this deficit based on statistics of the directional distribution of erosive wind speeds and their weighed transport capacities to calculate the effective lengths of the protected areas behind all LEs. Equations from the Wind Erosion Prediction System (WEPS) were used for this purpose, which calculate the wind speed reduction behind wind barriers as function of its height and its porosity.

The method is embedded in an ArcGIS toolbox and calculates the sheltering effects of all LE automatically. It is displayed in 5 reduction classes in a map which can be combined with soil or erodibility maps. The applicability is possible from individual, event based cases to an average assessment based on long-term data.

## Method details

### Input data

#### Wind speeds and directions

The wind data analysis should be based on values of the standard wind speed measuring height of 10 m. Data from lower heights can also be used, but the selected threshold value of the erosive wind speed must then be adjusted accordingly. The conversion can be carried out using standard boundary layer meteorological methods [7].

The wind data analysis is a preparatory step, which calculates the needed input data for the ArcGIS toolbox to set the virtual shadows in direction and length. The lengths of the shadows are derived from the frequency distribution of measured wind speeds (u) and their directional distribution. Wind speeds are classified in the range 0 - 20 m s^-1^ with a class width of 1 m s^-1^ for each considered direction. There are options for 8, 16 or 36 directions by the provided Excel-files. The Excel-sheets can be edited so that any other configuration can also be used. Setting a threshold value for the erosive wind speed (u_t_) deletes all values below it and the frequency distributions for each wind direction are recalculated with the remaining values. The frequencies are then multiplied with the transport capacity (q) of each wind speed using Eq. (1) [8] and summed up for each direction. Eq. (1) roughly describes the intensity of sediment transport as a function of the wind speed (u) above a threshold value (u_t_).

(1) $$q=\left(u-u_{t}\right)u^{2}$$

The calculation of the effective protection length (= maximum length of the shadows) for each wind direction is based on equations from the Wind Erosion Prediction System to simulate wind barrier effects (WEPS, Hagen and Fox, 2020; [9]). The equation calculates the decrease and increase of the wind speed behind a LE as multiples of its height. At this point, the porosity (p) of the windbreak can be entered as an additional parameter in the appropriate sheet of the Excel-file. This parameter can only be assigned once and is then valid for all LE. An average value is sufficient for the evaluation of long-term mean values; if the method is used on an event-related basis, it is possible to react to the different foliage in winter or summer.

(2) $$f_{u}=1-exp\left(-mx^{2}\right)+nexp\left(-0.003{\left(x+s\right)}^{t}\right)$$

where *f*_u_ wind reduction factor *x* distance from the structure element in multiples of height.

The coefficients m, n, s and t depend on porosity (p) and are calculated as follows

(3) $$m=0.008-0.17p+0.17p^{1.05}$$

(4) $$n=1.35exp\left(-0.5p^{0.2}\right)$$

(5) s=10(1−0.5p)

(6) t=3−p

**Fig. 1:**
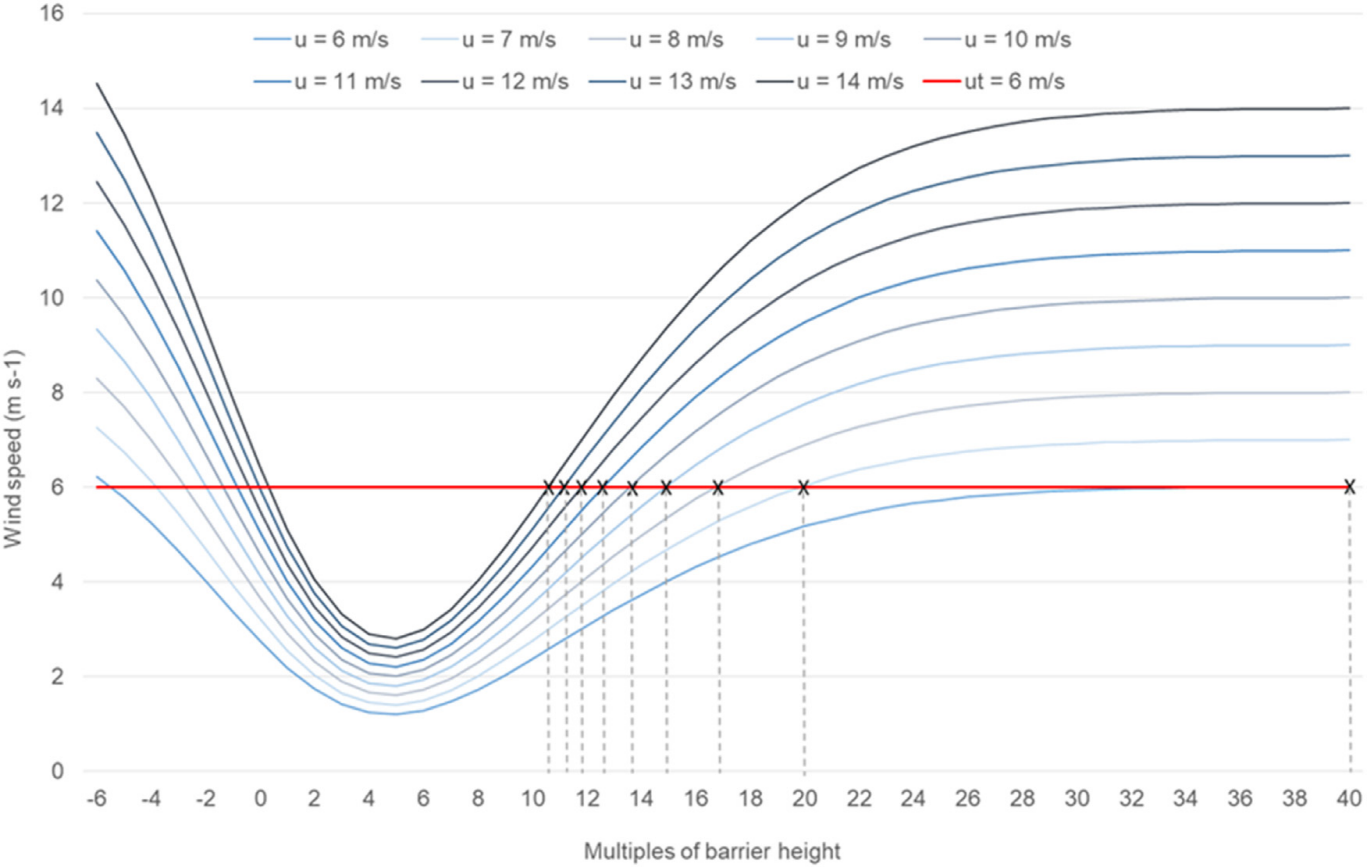
Wind speed reduction in front and behind a landscape element for all considered wind speeds above a threshold wind speed of 6 m s^-1^; effective shelter distance for each wind speed is reached at the intersection point with the threshold wind speed.

The wind reduction is then calculated with Eq. (2) for all wind speeds between 4 and 14 m s^-1^ in steps of 1 m s^-1^ and for distances between −6 to 40 in multiples of the heights (Fig. 1). The upper limit is set by the maximum measured wind speed in the area under consideration, which is in Brandenburg 14 m s^-1^ as an hourly average. The already selected threshold value of the wind speed of 6 m s^-1^ also sets the lower limit here. For each wind speed above the threshold the program selects the distance in multiples of the height at which the wind speed is below the threshold value for the first time (crossing the u_t_ – line in Fig. 1). Between two wind speed classes the mean to the next higher class is chosen. This is the effective length of protection of the landscape elements for the corresponding wind speed. Combining the effective length of protection with the relative transport capacity of every wind speed class results in the weighted effective length of protection for each direction. Finally, a further weighting is carried out between all directions by using the direction-dependent transport capacities. The direction with the highest transport capacity receives the value 1 and all others are shortened by their relative share per factor (Fig. 3, left). The total length is then divided into 5 equal parts, the length of each is then used to calculate the parameters required to display the virtual shadows (azimuth and altitude). These are then stored in an extra sheet of the Excel file, which the ArcGIS toolbox WERA then accesses.

**Fig. 2:**
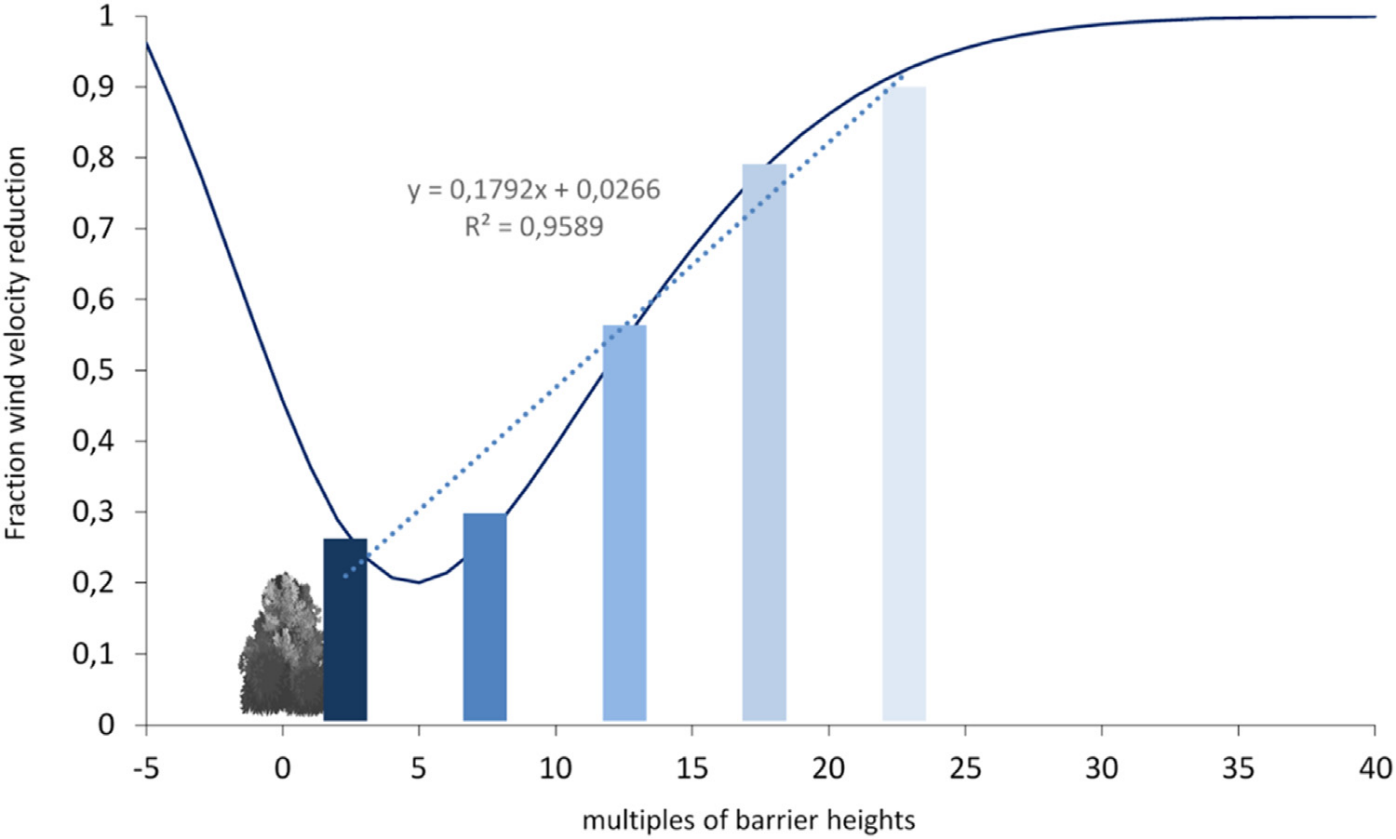
Transformation of the function of wind speed reduction (Eq. (2)) into the 5 protection zones by taking the averages of the used classes from 0 to 5, 5 to 10, 10 to 15, and so on.

The calculated shelter distances by Eq. (2) has to be transformed into the 5 protection classes used in the German Standard DIN 19706. For this purpose, the averages were calculated for each class (Fig. 2), which can be represented very well by linear regression and thus justifies the division into 5 classes of equal size. This appears to be sufficiently accurate for our requirements.

**Fig. 3:**
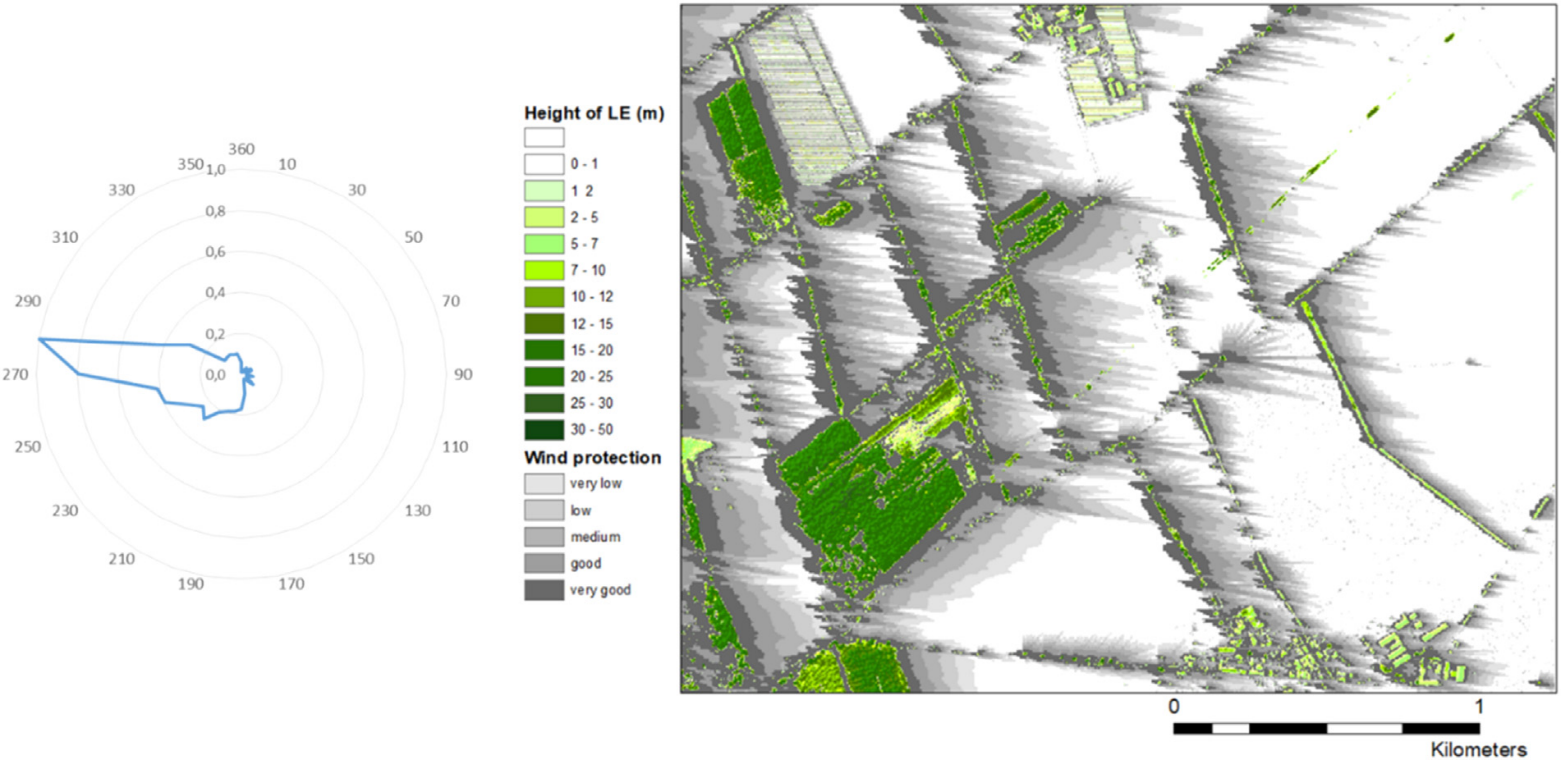
Fractions of relative transport capacities of erosive winds from all directions (left wind rose) and landscape section with the appropriate protection zones divided in the 5 protection classes (grey) behind all landscape elements (green).

#### Landscape elements

The main parameter of any LE to derive the wind reducing effect is its height. This information can be obtained in various ways, best from uniform data available for the entire area under consideration. The following are suitable for this purpose: digitized maps, own measurements or also by means of laser scanning or photogrammetry. In the case of using maps, where LE are in vector format, a height must be assigned to each class. The transformation of this layer into a raster layer shows the height of each pixel as z-value. This makes it possible to set virtual shadows in a GIS. Raster based methods of height determination, as laser scanning or photogrammetry, can be used immediately. Since these methods are all based on a top view of the landscape, parameters that can be revealed from the side view, such as the density of LE, are difficult to derive. However, this can be taken into account via the effect on the wind speed (Eqs. (2)-(6)). It is only important that all pixels, not representing an LE are given the value zero. We set the grid size to 2 × 2 m, so that even LE with a height of 1 m show effects.

#### Soil erodibility data

One of the inputs is a raster map of the soil erodibility. In our example it is derived from soil texture and soil organic matter content. We used the vectorised maps of the German soil quality appraisal (Bodenschätzung, scale 1:10,000) and the Medium Scale Agricultural Mapping (MMK, scale 1:25,000), which were combined and converted into a grid. The classification is based on the German standard “DIN 19706″ and the potential wind erosion risk is presented in the usual 6 classes from no risk to very low, low, medium, high and very high. The data resulting from these step for further processing are the numbers from 0 to 5, so that any other evaluation schemes for soil erodibility can also be used for this purpose, resulting in the same range of classes. The pixel size of that grid should be equal with the grid of the landscape elements that means in our example a pixel size of 2 × 2 m.

#### GIS toolbox WERA

The toolbox consists of five modules which can be processed separately and provides partial results after each processing step. These can then be used in their current form or for the further calculation.

**Fig. 4:**
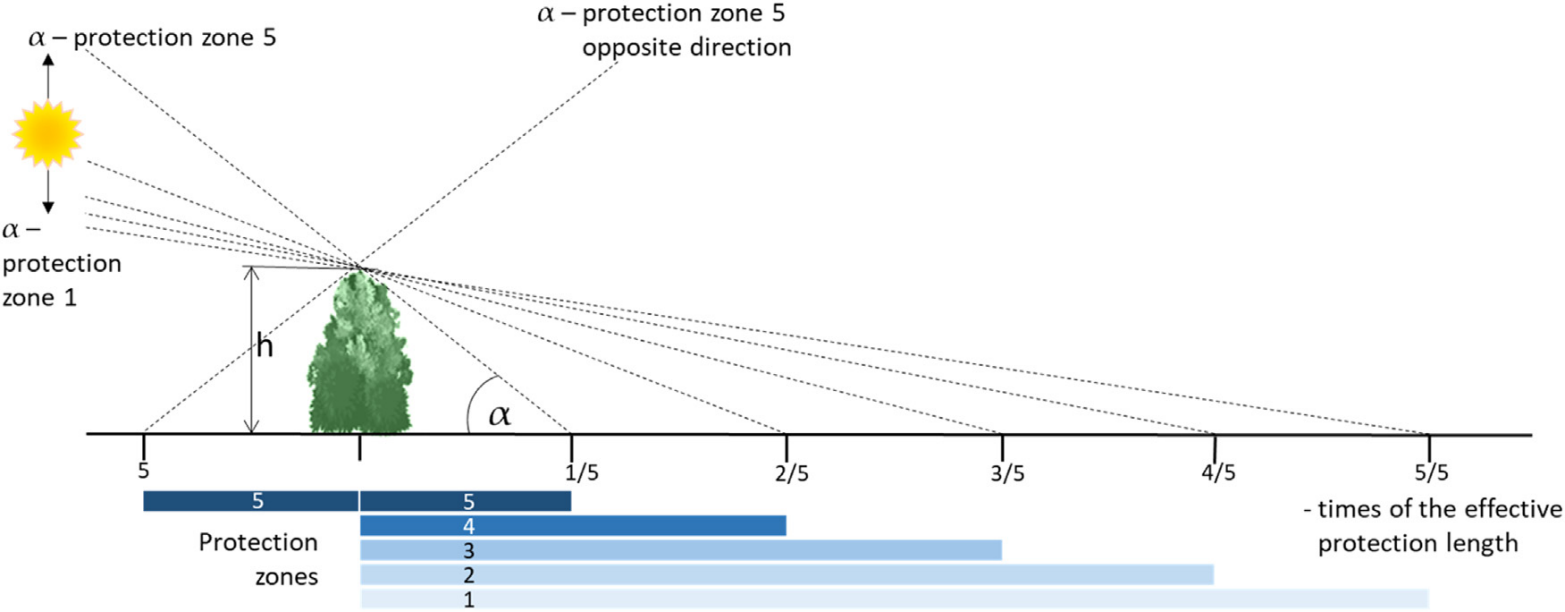
Scheme of the shadow setting by the GIS-command “hillshade” to create protection zones in front and behind landscape elements.
***Module 3*** overlays first all calculated protection zones of one direction in one layer and then all layers of the selected number of directions (8, 16, 36) by taking the maximum value of the protection zones for each cell. The result are the wind protection classes for the entire area in one final layer.
***Module 4*** combines the maps of the wind erodibility of the soils with wind protection classes with the following matrix:

***Module 1*** has the purpose to clear the arable land of obstacles that are not part of the landscape structure. This step is particularly necessary when the heights are derived from the Digital Surface Model (DSM) and the Digital Elevation Model (DEM) [10], as haystacks or hay bales inside the fields were also scanned and the subtraction of the DEM from the DSM does not always result exactly in the value 0. The mask for this is provided by the Digital Field Block Cadastre (DFBK 2023), which contains the current field blocks as shapes with high positional accuracy. Everything within the field blocks is set to the value 0. Output is the cleaned raster map of the LE which is the input for Module 2.
***Module 2*** calculates the wind shadows of the landscape elements. Depending on the number of wind directions considered (8, 16 or 36), Module 2 accesses the corresponding Excel table and uses the values calculated there for azimuth (column “Azimuth”) and altitude (column “Altitude”) to calculate the shadows by the command “Hillshade”. After loading the raster data of the cleaned LE, the virtual shadows are calculated for each pixel. Five shadows with increasing lengths are created in one direction and one in the opposite direction (Fig. 4). Each shadow is filled with the appropriate number (column “Constant”) of the protection zone (1 – 5) and is saved in a separate file.

**Table:** 

This results in a map of the susceptibility of soils to wind erosion, which takes wind protection by LE into account.

***Module 5*** allows to analyse the wind erosion risk within certain structures. The raster map generated in Module 4 is queried with the structures of the field blocks. As a result, field blocks with an area of a certain, selectable percentage in the very high wind erosion risk class are selected and the appropriate field blocks are marked. Other structures can also be set up here, such as area of a farm, municipal or district areas.

### Method validation

A validation has not yet taken place. The method is based on correlations that have been studied for decades and are well proven. A validation would be best realized after wind erosion events, when the areas of transport, saturation and deposition are clearly visible on a field or in the landscape.

## Limitations

Our approach explicitly excludes the roughness of the terrain, as Brandenburg is located in the northern German lowlands with the highest elevation of about 200 m by a total area of 30,000 km². Also, factors of short-term changes, such as soil moisture and soil roughness by management, are not taken into account.

## Ethics statements

No ethical statements have to be declared.

## Supplementary material and/or additional information

All data, such as Excel file ArcGIS-Toolboxes, as well as a user manual are available on request.

## CRediT authorship contribution statement

**Roger Funk:** Conceptualization, Methodology, Project administration, Funding acquisition. **Lidia Völker:** Methodology, Software, Validation, Data curation, Visualization.

## Declaration of competing interest

The authors declare that they have no known competing financial interests or personal relationships that could have appeared to influence the work reported in this paper.

## Data Availability

Data will be made available on request.

## Appendix Supplementary materials

Image, application 1

Image, application 2

Image, application 3

Image, application 4

Image, application 5
